# Preliminary Study on the Expression and the Clinical Significance of CD133 in Peripheral Blood of Patients with Gastric Adenocarcinoma

**DOI:** 10.1155/2014/245329

**Published:** 2014-02-06

**Authors:** Ju-gang Wu, Ji-wei Yu, Rui-qi Lu, Shou-lian Wang, Xiao-chun Ni, Lin-hai Zheng, Bo-jian Jiang

**Affiliations:** 1st Department of General Surgery, Shanghai 3rd People's Hospital, School of Medicine, Shanghai Jiao-Tong University, No. 280 Mohe Road, Shanghai 201900, China

## Abstract

*Background*. Significances of CD133 mRNA in peripheral blood mononuclear cells (PBMCs) of gastric adenocarcinoma (GC) patients were investigated. *Methods*. Correlations of CD133 mRNA expression in PBMCs on clinicopathological parameters or CD133 protein expression were analyzed. Receiver operating characteristic curve according to bright scale value (BSV) of CD133 mRNA was used to group patients for prognosis analysis. *Results*. BSV of preoperative CD133 mRNA in PBMCs in GC was significantly higher than that in volunteers or in GU. Invasive depth or metastatic lymph node number for higher BSV of preoperative CD133 mRNA and invasive depth or lymphatic vessel invasion for higher BSV of postoperative CD133 mRNA in the PBMCs were identified. Patients with CD133^+^ expression in primary lesion had a significantly higher expression of preoperative CD133 mRNA in the PBMCs. The expression of preoperative or postoperative CD133 mRNA in PBMCs related positively to CD133 mRNA expression in primary lesion. Patients with higher expression of preoperative or postoperative CD133 mRNA shared significantly shorter survival compared with that in lower expression group. Conclusion. Higher levels of preoperative or postoperative CD133 mRNA in PBMCs of GC correlated positively to the lymphatic metastasis and the BSV of CD133 mRNA in primary lesion, indicating the poorer survival.

## 1. Background

Gastric cancer (GC) with only about 20% of overall 5-year survival rate until now is one of the most common forms of cancers worldwide because it is usually diagnosed too late, especially in the developing countries such as China, due to defections of typical symptoms, effectively imaging means and specific tumor biomarkers. Therefore, such advanced cases with later stages of TNM are often unsuitable to receive a curative surgery [1]. Recently, a lot of studies suggested that a small population of cancerous cells named as so-called cancer stem cells (CSCs) with unique self-renewal properties and malignant potentials existed in some sorts of solid tumors [2–4]. As reported, these CSCs could be sorted by flow cytometry or by immunomagnetic separation (IMS) and had been demonstrated to share the stronger abilities of tumorigenicity *in vivo *and cloning sphere formation *in vitro* [3–5]. As well known, one of the sorting markers frequently used in the separation/enrichment to these cancerous cells with stemness characteristics in gastrointestinal neoplasm is the cell surface protein nominated as CD133/prominin-1 [5–7].

The accumulated evidence has shown that CD133 as a meaningful marker in the surface of circulating endothelial progenitor cells (CEPs) may play greatly important roles in tumor angiogenesis [8–10]. As reported, the CEPs probably differentiated from the matured vascular endothelial cells or from the activated circulating endothelial cells (CECs) [10] and the CEPs had a greatly proliferating ability and could furthermore form new vasculature [11, 12]. Recently, some investigations found that, in patients with some advanced malignancy, the high level of CEPs in the peripheral blood mononuclear cells (PBMCs) could be detected [8–10]. On the other hand, compared with a rate as high as 90% of CEPs subpopulation found in xenograft models [13], the rate of CEPs subpopulation was only from 1% to 12% in different sorts of cancers in human being [14].

CD133 identified as expressing highly in human GC tissues [15–18] may be a potential therapeutic target applying specific blocker conjugating with antitumor drug. Smith et al. [19] found that a murine antihuman CD133 antibody conjugated to a potent cytotoxic drug could effectively inhibit the growth of GC cell and significantly delay tumor growth in SCID mice. Our previous study [16] showed that higher expressive level of CD133 mRNA was associated to lower Ki-67 LI and severer lymphatic metastasis. The expressive level of CD133 mRNA could play an appropriate role to reflect the status of lymph node metastasis and proliferation of GC. As prognostic analyses by the application of CSCs marker of CD133, CD133^+^ patients with GC shared poorer prognosis [17, 18, 20, 21]. In these investigations [16–18, 20, 21], the positivity expression of CD133 protein was closely associated with larger tumor, later stage of TNM, severer lymphatic metastasis, and poorer survival of GC. In this preliminary study of ours, the expression level and the clinical significance of CD133 mRNA in PBMCs of patients with GC would be detected and analyzed as a preliminarily valuable trial to find some clues as the conveniently means before and after operation, indicating clinicopathologic status and prognosis of GC.

## 2. Methods

### 2.1. Clinical Data

Between January 2008 and December 2010, 70 patients with gastric adenocarcinomas (GC) from the Department of General Surgery, Shanghai 3rd People's Hospital, School of Medicine, Shanghai Jiao-tong University were enrolled in this study. All patients underwent a curative gastrectomy (*D*_2_, *R*_0_/*R*_1_) and none of them received preoperative chemotherapy or radiotherapy. The patients in this study included 45 men and 25 women, ranging in age from 29 yr to 83 yr, with a median age of 65 yr. Total gastrectomy was performed in 23 patients, distal subtotal gastrectomy in 41 patients, and proximal subtotal gastrectomy in 6 patients. The pathological examination after surgery showed 13 cases of early stages and 57 cases of advanced stages including 5 cases of fungating type, 19 cases of ulcerative type, 23 cases of invasion ulcerative type, and 10 cases of diffuse infiltrative type. All clinicopathological profiles were evaluated in accordance with the 14th edition criteria of the Japanese Gastric Cancer Association [22]. The deadline of following-up to patients was on Dec 31, 2012 and the mean time of follow-up was 27.01 ± 11.65 months. During the follow-up period, 23 patients died of recurrence or metastasis and 2 patients died of other diseases of non-cancer.

Ten healthy volunteers (6 men and 4 women, age range 35 yr−58 yr) and 10 patients with gastric ulcer (GU) (7 men and 3 women, age range 30 yr−63 yr) were registered as negative controls. This study was approved by the ethical committee of Shanghai 3rd People's Hospital, School of Medicine, Shanghai Jiao-tong University before its start. Written consent for this investigation and this publication was obtained from all of patients or patients' relatives.

### 2.2. Sample Preparation

For each person with GC or GU or healthy volunteer, 4 mL of peripheral blood was, respectively, collected before operation and at 1 week after surgery before breakfast.

Half sample from the fresh tissue of primary lesion of GC was immediately stored in −80°C and another half was fixed in 10% formalin solution and then embedded by paraffin for examinations of histology and immunohistochemistry. Noncancerous gastric tissues (NCGT) taken in the site of >5 cm distance adjacent to primary lesion and identified by pathological observation were used as negative controls to the expressions of mRNA and protein of CD133 taken from primary lesion of GC.

### 2.3. Separation of PBMCs

Peripheral blood sample was collected in a sterile tube with 3.8% natrium citricum (w/v). Firstly, the peripheral blood sample was diluted as 1 : 1 with PBS. Before making Percoll solution (Amerham Biosciences AB, Uppsaia, Sweden) warmed at room temperature, same volumes of 72% Percoll solution and 63% Percoll solution were transferred to a 15 mL centrifuge tube from the bottom to the top. Then, the diluted blood was carefully layered over the Percoll solution. The entire samples were centrifuged at 500 g under 4°C for 25 min. Secondly, mononuclear cells, that is, PBMCs, between two layers of different concentration of Percoll solution were extracted carefully and then washed once slightly with PBS. Finally, PBMCs were immediately added to RNAiso Plus Reagent (TaKaRa Biotechnology, Tokyo, Japan) and stored at −80°C until future use [12].

### 2.4. Reverse Transcriptase-Polymerase Chain Reaction (RT-PCR) Detection

Total RNA was extracted from PBMCs or 80–100 mg frozen GC tissue treated with RNA PCR kit (TaKaRa Biotechnology, Tokyo, Japan) following the manufacturer suggested protocols. Oligo dT-Adaptor Primer was used with AMV reverse transcriptase XL for cDNA synthesis from 0.5 micrograms of total RNA. PCR was conducted with TaKaRa *Ex Taq* HS DNA polymerase (TaKaRa Biotechnology, Tokyo, Japan) in 50 *μ*l reaction volumes. Various Primers (synthesized by Sangon Technology, Shanghai, China) used in this study included GAPDH (sense, 5′-ACGGATTTGGTCGTATTGGGCG-3′; antisense, 5′-CTCCTGGAAGATGGTGATGG-3′), with a product length of 197 bp, and CD133 (sense, 5′-TTACGGCACTCTTCACCT-3′; antisense, 5′-TATTCCACAAGCAGCAAA-3′), with a product length of 172 bp. The reactions were conducted for GAPDH as the internal control under the following conditions: initial denaturing step at 95°C for 1 min, 28 cycles of 95°C for 1 min, 55°C for 1 min, and 72°C for 1 min, followed by 72°C for 10 min; for CD133 as the following conditions: initial denaturing step at 94°C for 2 min, 35 cycles (28 cycles for sample from frozen gastric cancer tissues) of 94°C for 30 seconds, 57°C for 30 seconds, and 72°C for 30 seconds, followed by 72°C for 10 min. The amplified products by MyCyclePCR (Bio-Rad Laboratories, CA, USA) were separated on a 1.5% agarose gel (Gene Tech, Shanghai, China) by electrophoresis apparatus (Bio-Rad Laboratories, CA, USA). Digital images to expose the occurrence of CD133 mRNA as a white target strip were captured on a gel documentation system (UNIVERAL HOOD II, Bio-Rad Laboratories, Segrate, Italy). Imaging assessments to measure the brightness scale value (BSV) of CD133 automatically from the white strip and to compare the relative ratio between CD133 strip and control strip were carried out by Quantity One 1-D analysis Software (The Discoveries Quantity One 1-D Analysis Software Version 4.5, Bio-Red Laboratories, CA, USA) [16].

### 2.5. Immunocytochemical Staining

Endogenous peroxidases were blocked using 3% H_2_O_2_ solution for 30 min followed by incubation with blocking serum (Santa Cruz Biotechnology, CA, USA). Incubation with primary antibody of CD133/1 (1 : 40, Miltenyi Biotec GmbH, Bergisch Gladbach, Germany) or CK20 (1 : 40, Changdao Technology, Shanghai, China) was performed for 1 h at room temperature. And then, immunodetection was performed by ABC staining system (Santa Cruz Biotechnology, CA, USA). Cells were couterstained with haematoxylin to show the nuclear in detail. *Negative controls* were reacted with *PBS* in place of primary antibody of CD133 or CK20 [16, 23].

### 2.6. Clinicopathological Parameters

Gender, age, tumor size, depth of invasion, LNM, TNM stage, tissue differentiation, Lauren type, blood vessel invasion, lymphatic vessel infiltration, and metastatic lymph nodes ratio (MLR) were used as clinicopathological parameters in this study. All clinicopathological profiles were evaluated according to the criteria of the Japanese Gastric Cancer Association [22]. In this context, the maximum diameter of primary lesion would be used to assess the size of tumor and MLR formula would be as follows: MLR = (the number of metastatic lymph nodes/the number of total detected lymph nodes) × 100% [24]. ROC curves were used to assess the accuracy of the BSV of CD133 mRNA to predict the prognosis [22]. According to Youden's index, the maximum *J* value was applied as grouping standard for survival analysis. *J* = Sensitivity + Specificity − 1 [25].

### 2.7. Statistical Analysis

The software of SPSS13.0 for Windows version (IL, USA) was used to analyze the data of this study. In addition, measurement data were tested by nonparametric statistics (*k* independent samples test or 2 independent samples test) and analyzed with relative correlation of Spearman's rho. Multivariate analysis was made by logistic method. Patient survival was analyzed using the Kaplan Meier product limit method. The log rank test was used to evaluate the difference between groups. The level of statistical significance was defined as *P* < 0.05.

## 3. Results

### 3.1. Expression of CD133 mRNA in PBMCs before Operation

The expression of CD133 mRNA was present in 20% (2/10) of healthy volunteers, 30% of patients (3/10) with GU, and 90% (63/70) of patients with GC. The average BSV of CD133 mRNA was 0.029 ± 0.060 (0~0.151) in the group of healthy volunteers, 0.059 ± 0.099 (0~0.266) in the group of patients with GU, and 0.262 ± 0.149 (0~0.746) in the group of patients with GC. Therefore, the significant differences in the average BSV were seen obviously among these three groups by statistic analysis (*χ* ^2^ = 26.724, *P* < 0.001). By two independent samples tests of nonparameter of statistic analysis, it was found that the average BSV of CD133 mRNA in the group of patients with GC was significantly higher than that in the group of volunteers (*Z* = −4.132, *P* < 0.001) or in the group of patients with GU (*Z* = −3.688, *P* < 0.001), respectively, but there was no statistic difference between the group of volunteers and the group of patients with GU (*Z* = −0.695, *P* = 0.487). The above-mentioned results indicated that the measure of CD133 mRNA from PBMCs could be worth of further investigations as a diagnostic value in GC if combined with the examination of gastric fiber or radiological imaging observation.

### 3.2. Correlation of CD133 mRNA Expression in PBMCs before Operation with Clinicopathological Parameters

The relationships between the BSV of CD133 mRNA in the PBMCs before operation with the clinicopathological parameters of GC were analyzed. The higher BSV of CD133 mRNA in the PBMCs of patients with GC before operation was associated with the poorer differentiation (*P* = 0.007), the lymphatic vessel invasion (*P* = 0.006), the deeper invasion (*P* = 0.003), the lymph nodes metastasis (LNM) (*P* < 0.001), and the later stages of TNM (*P* < 0.001) (Table 1).

Totally, 1285 lymph nodes in 70 patients with GC were examined in this research, with an average number of 18.36 ± 7.16 nodes per case (ranging from 16 to 52 per case). In all, total metastatic lymph nodes were 359, with an average number of 5.13 ± 6.711 metastatic lymph nodes per case (ranging from 0 to 27 per case). At the same time, the average MLR in 70 patients of GC was (24.68 ± 30.03)% (ranging from 0 to 92.86%). As shown in Table 2, multivariate risk analysis showed that invasive depth or metastatic lymph node number was an independent impact factor for the higher expression of preoperative CD133 mRNA in PBMCs.

Relative analysis revealed that the higher expression level of CD133 mRNA in the PBMCs before operation related positively to the higher MLR (*r* = 0.399, *P* = 0.010) and metastatic lymph node number (*r* = 0.372, *P* = 0.002) but did not relate positively to the total number of examined lymph nodes (*r* = 0.019, *P* = 0.876).

### 3.3. Comparison of Preoperative CD133 mRNA Expressions in PBMCs with CD133 mRNA Expression in Primary Lesion of GC

In this study, the expression of CD133 mRNA was also identified to present in all 70 samples from frozen tissues of primary lesion of GC (70/70), which was significantly higher than that in NCGT (12.857% (9/70), *P* < 0.001). The BSV (0.344 ± 0.152 (0.091~0.734)) of CD133 mRNA in primary lesion was significantly higher than that in the NCGT (0.040 ± 0.093 (0~0.124) (*Z* = −7.789, *P* < 0.001)).

**Figure 1:**
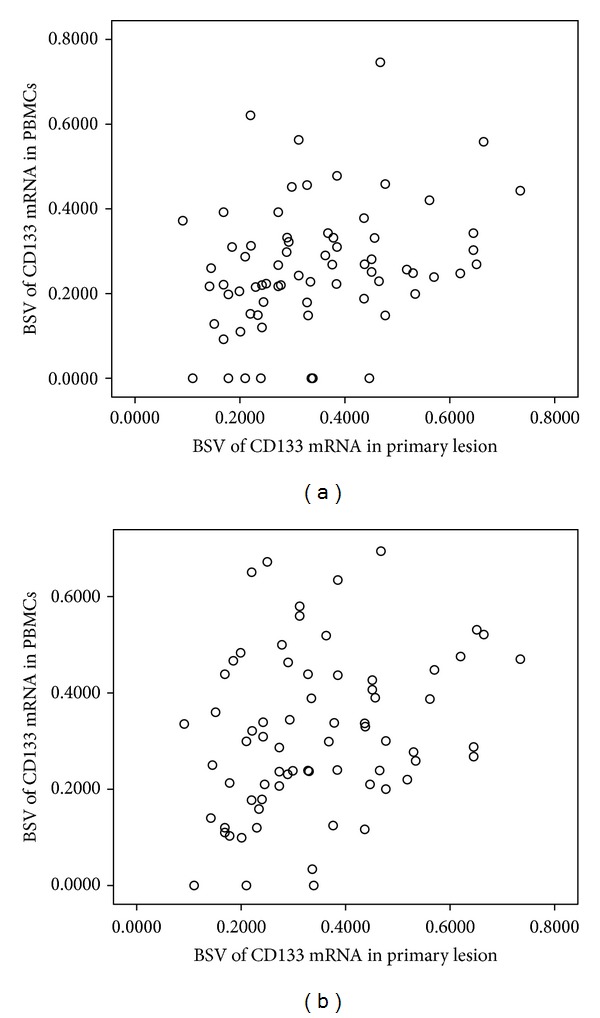
Relation of the BSV of preoperative or postoperative CD133 mRNA in PBMCs on the BSV of CD133 mRNA in primary lesion. (a) Preoperative; (b) postoperative.

Concurrently, the relative analysis revealed that the expressive level of preoperative CD133 mRNA in the PBMCs related positively to the expressive level of CD133 mRNA in the primary lesion of GC (*r* = 0.310, *P* = 0.009) (Figure 1(a)), indicating that the increased level of CD133 mRNA in the PBMCs before operation was positively corresponding to the higher level of CD133 mRNA in primary lesion of GC.

### 3.4. Comparison of CD133 mRNA Expression in PBMCs before Operation and Immunohistochemical Expression of CD133 Protein in Primary Lesion of GC

**Figure 2:**
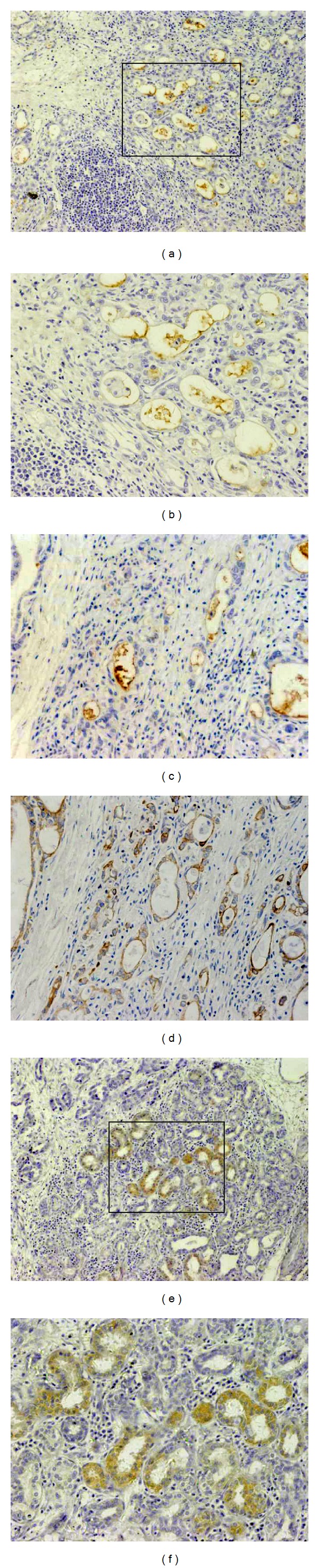
Morphologic characteristics of CD133 positive cells in primary lesion by IHC staining. (a) CD133^+^ cells located in the wall of crypts or in the inner compositions of the crypts with GC (×100). (b) Almost all the stained CD133 proteins located in the surface of cell membranes (×200). (c) CD133^+^ cells located in the inner compositions of crypts stained by CD133 monoclonal antibody. (d) Consecutive sections stained by CK20 monoclonal antibody. (e) Some special CD133^+^ cells cytoplasm was stained and these CD133^+^ cells formed the crypts in tissue section (×100). (f) The size and shape of these CD133^+^ cells were observed in larger magnification. These crypts in tissue sample of GC liked morphologically to the normal crypts of stomach (×200).

Immunohistochemical staining showed that the positive expression rate of CD133 protein in primary lesion of GC patients was 32.857% (23/70). Some CD133 positive cells located in the wall of crypts with GC (Figures 2(a) and 2(b)) and others occurred in the compositions of the crypts which were not stained by CK20 in a serial section (Figures 2(c) and 2(d)). Almost all of these stained CD133 proteins were located in the surface of cell membranes. Interestingly, some CD133 protein positive cells formed the crypts, in which the cytoplasm of these cells was positively stained in the serial sections of 1 patient with GC (Figures 2(e) and 2(f)).

Furthermore, 23 patients with positive expression of CD133 protein had a significantly higher expression level of preoperative CD133 mRNA (0.331 ± 0.138) in the PBMCs than that (0.229 ± 0.144, *Z* = −2.490, *P* = 0.013) in the group of patients without expression of CD133 protein in primary lesion.

### 3.5. Clinical Significances of High Expression of CD133 mRNA in PBMCs after D_2_ Resection in GC

The CD133 mRNA expressions in the group of patients with GC at 1 week after D_2_ resection were detected in 94.29% (66/70) cases while the average BSV of CD133 mRNA was 0.313 ± 0.165 (0~0.694), which was significantly higher than that in the group of patients with GC before operation (0.262 ± 0.149, *t* = −2.392, *P* = 0.021). The higher BSV of postoperative CD133 mRNA in the PBMCs of patients with GC was also associated with the lymphatic vessel invasion (*P* = 0.005), the deeper invasion (*P* < 0.001), the lymph nodes metastasis (LNM) (*P* = 0.001), and the later stages of TNM (*P* < 0.001) (Table 3). As shown in Table 4, multivariate risk analysis showed that invasive depth or lymphatic vessel invasion was an independent impact factor for the higher BSV of postoperative CD133 mRNA in PBMCs.

Concurrently, the expressive level of postoperative CD133 mRNA in the PBMCs related positively to the expressive level of CD133 mRNA in the primary lesion of GC (*r* = 0.284, *P* = 0.017) (Figure 1(b)).

### 3.6. Application of ROC Curve for the Analysis on the Correlation between the Expression of Preoperative CD133 mRNA in PBMCs and the Survival

**Figure 3:**
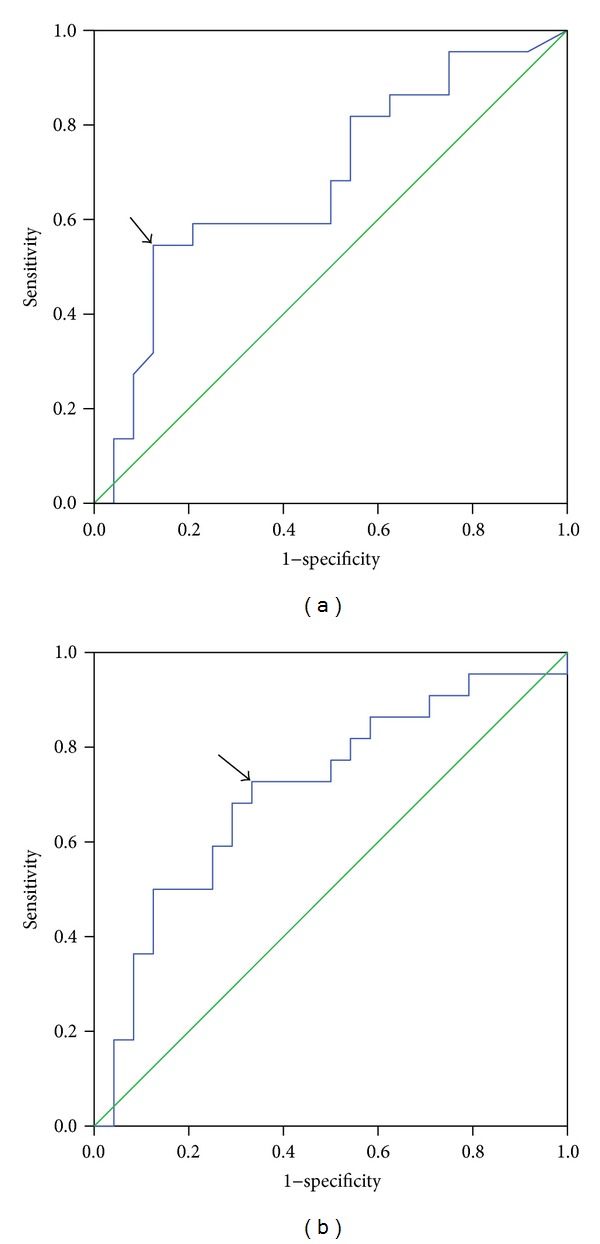
Application of ROC curve for the analysis on correlation of the BSV of preoperative or postoperative CD133 mRNA in PBMCs with the survival. (a) Preoperative; (b) postoperative. Arrows showed Youden's index.

After excluding those from the original 70 patients, who had died of other diseases or lost to follow-up in 3 years, the ROC curve was drawn according to the BSV of preoperative CD133 mRNA and the survival in the remaining of 46 patients (Figure 3(a)). The areas under the curves described above were 0.681 ± 0.081 (95% CI: 0.523~0.839, *P* = 0.036). Therefore, the expression of preoperative CD133 mRNA might be applied to predict the survival. The value of 0.316 as indicated by Youden's index was designated as cutoff of preoperative BSV of CD133 mRNA. Under these circumstances, the sensitivity and specificity were 54.5% and 87.5%, respectively.

**Figure 4:**
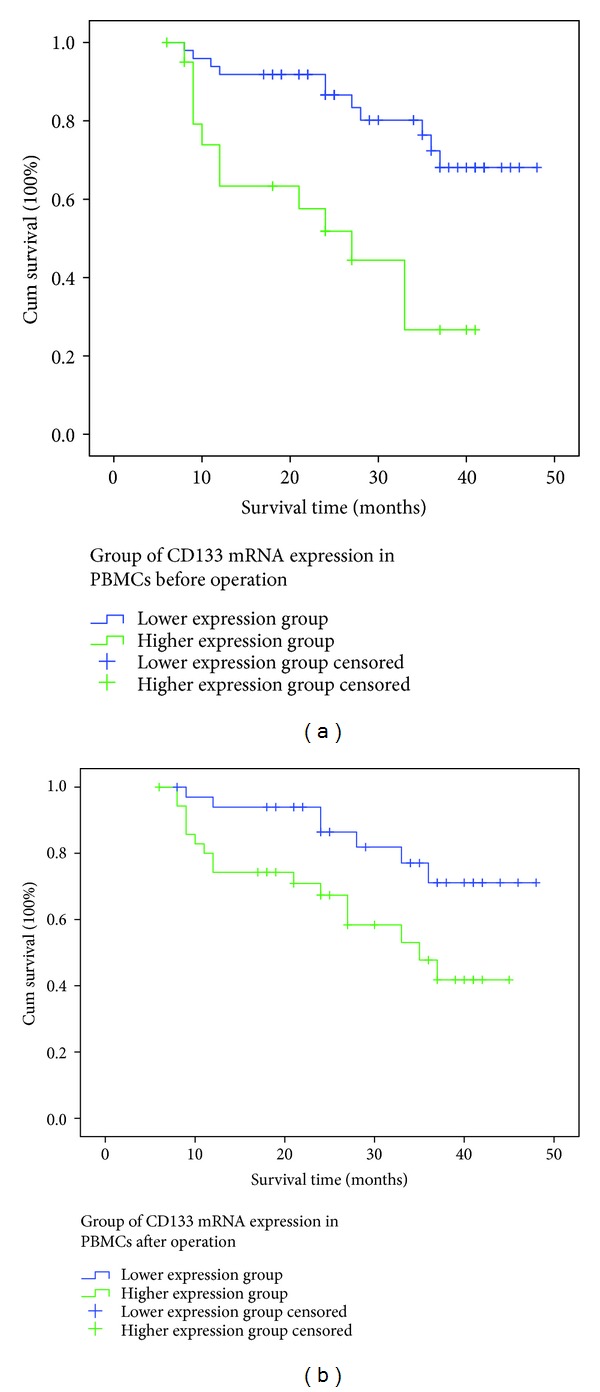
Relationship of prognosis with the preoperative and the postoperative BSV of CD133 mRNA in PBMCs. (a) Preoperative, *P* < 0.001; (b) postoperative, *P* = 0.018.

Then, 70 patients before surgery were also divided into two groups according to the value of 0.316 as a cutoff of BSV of preoperative CD133 mRNA. The lower expression of preoperative CD133 mRNA group (≤0.316) included 49 patients and the average BSV of preoperative CD133 mRNA in this group was 0.190 ± 0.094. And the higher expression of preoperative CD133 mRNA group (>0.316) included 21 patients and the BSV of preoperative CD133 mRNA in this group was 0.431 ± 0.113. Univariate survival analysis suggested that a significant difference in prognosis between these two groups could be identified (*χ* ^2^ = 12.670, *P* < 0.001). Postsurgery survival time was shorter in patients with a higher preoperative CD133 mRNA expression in comparison with that in lower preoperative CD133 mRNA expression group (Figure 4(a)).

### 3.7. Application of ROC Curve for the Analysis on the Correlation between the Expression of Postoperative CD133 mRNA in PBMCs and the Survival

Similarly, the ROC curve was drawn according to the BSV of postoperative CD133 mRNA and the survival of the remaining 46 patients (Figure 3(b)). The areas under the curves described above were 0.708 ± 0.078 (95% CI: 0.555~0.862, *P* = 0.016). Therefore, the expression of postoperative CD133 mRNA might be applied to predict the survival. The value of 0.293 as indicated by Youden's index was designated as cutoff of BSV of postoperative CD133 mRNA. Under these circumstances, the sensitivity and specificity were 72.7% and 66.7%, respectively.

Then, 70 patients after surgery were also divided into two groups according to the value of 0.293 as a cutoff of BSV of postoperative CD133 mRNA. The lower expression of postoperative CD133 mRNA group (≤0.293) included 34 patients and the average BSV of postoperative CD133 mRNA in this group was 0.177 ± 0.084. And the higher expression of postoperative CD133 mRNA group (>0.293) included 36 patients and the BSV of postoperative CD133 mRNA in this group was 0.441 ± 0.111. Univariate survival analysis suggested that a significant difference in prognosis between these two groups could be identified (*χ* ^2^ = 5.643, *P* = 0.018). Postsurgery survival time was shorter in patients with a higher postoperative CD133 mRNA expression in comparison with that in lower postoperative CD133 mRNA expression group (Figure 4(b)).

## 4. Discussion

CD133/prominin-1 as a transmembrane pentaspan protein was found to present in many solid tumors such as brain cancer [2], prostate cancer [3], and colon cancer [4]. O'Brien and his team [4] identified the tumor initiating cells (TICs) in human colon cancer, in which TICs possessed CD133^+^ expression. The inoculation of minor number of CD133^+^ cells was able to initiate the formation and the growth of tumor in *in vivo* experiment and these CD133^+^ cells could maintain themselves and differentiate and reestablish tumor heterogeneity upon serial xenogeneic transplantation. Moreover, the CD133 marker could also be applied to identify CEPs specifically. It was also reported that CD133 might take an important role in angiogenesis of cancer [13, 26, 27].

In previous study of ours [16], it was found that the CD133 mRNA expression in the tissue samples from the primary lesion of GC were present in all detected cases with GC. The BSV of CD133 mRNA in the primary lesion was significantly higher than that in the NCGT (*P* < 0.001). On the other hand, the expressive level of CD133 mRNA in the tissue samples from the primary lesion of GC was positively related to the MLR and the number of metastatic lymph nodes. Collectively, these results strongly suggested that CD133 might play a key role in the invasion and the metastasis of tumor, spatially in the process of LNM of GC.

Based on the progression in the studies on CD133 in gastrointestinal tumors, we focused on the expression and the clinical significances of CD133 mRNA from PBMCs isolated from peripheral blood of GC patients. The technique of RT-PCR which was a simple, rapid, and minimally invasive method was applied to check the CD133 mRNA in PBMCs. The relationships of the average level of CD133 mRNA in PBMCs with various clinicopathological parameters and the expressive level of CD133 mRNA in primary lesion were analyzed also. Encouragingly, our results suggested that CD133 mRNA expression was elevated in PBMCs from patients with GC before operation, which was associated with poorer differentiation, occurrence of lymphatic vessel invasion, deeper invasion, later stages of LNM or MLR, much more number of metastatic lymph nodes, and later stages of TNM. The multivariate risk analysis showed that patients with deeper invasion or more metastatic lymph nodes suffered a higher BSV of preoperative CD133 mRNA in PBMCs. These results of ours were similar to other scholar's report in the occurrence of bone metastasis [25]. In their report, CD133 mRNA was elevated in PBMCs from patients with bone metastases and its higher level was an independent prognostic factor. On the other hand, as an elevated CEPs marker, the higher level of CD133 mRNA had been used to predict colon cancer recurrence [28]. Similarly, the expressive level of CD133 mRNA in PBMCs also related positively to the expressive level of CD133 mRNA in primary lesion tissue samples of GC as demonstrated in this experiment of ours. Hence, the simultaneous occurrences of CD133 mRNA both in peripheral blood and in primary lesion might deduce some mechanism of GC metastasis.

IHC staining showed that some CD133^+^ cells were located in the wall of crypts in the primary lesion of GC. Other was inner the crypts, which CD133^+^ cells were not stained by IHC staining of CK20. CK 20 as a component of the cytoskeleton of endothelial cells is expressed only in differentiated gastric endothelium, which means that these CD133^+^ cells may be the undifferentiated or the poorer differentiated tumor cells like some specificity and activity of CSCs/TICs [23]. As revealed by this study of ours, almost all of the stained CD133 proteins were located in the surface of cell membranes. But some CD133^+^ cells were proved to form the crypts and the cytoplasm with CD133^+^ particles was identified in adjacent sections of primary lesion in one patient with GC. In order to confirm this interesting phenomenon, serial sections for CD133 IHC were performed furthermore. The observation on these tissue sections suggested that the size and shape of these CD133^+^ cells were similar. Even the nuclei of CD133^+^ cells were in similar shape and the mitosis of these cells could fairly be identified yet. These crypts in tissue sample of GC liked morphologically to the normal crypts of stomach. So, it might support the hypothesis that CD133 was the novel marker of “gastric CSCs.” However, further researches to deduce this hypothesis are certainly necessary.

In this research of ours, we found that the level of CD133 mRNA in PBMCs of patients with GC postoperatively was higher as compared with preoperatively. Moreover, patients who suffered from deeper invasion were inclined to have a higher level of CD133 mRNA expression after surgery (*P* = 0.039). This result was similar to some other reports [28, 29]. Iinuma et al. [28] found that the patients with Dukes' B or C of colorectal cancer, who had undergone curative surgery, could possess a higher level of CD133 mRNA in PBMCs. Those patients also suffered from much more risks in cancer recurrence and in worse overall survival or disease-free survival. Nakamura et al. [29] demonstrated that the CD133 mRNA expression could be detected in the peritoneal washings in colorectal cancer after curative resection. The higher level of CD133 mRNA in the peritoneal washings indicated a significant correlation with the severer LNM and the later stages of TNM. The overall survival rate and the survival rates with peritoneal recurrence free in the patients with CD133 mRNA positivity were significantly lower. From our data in this study, patients with *T*_3-4_ were 26 cases (52%) and *T*_4_ were 19 cases (38%) especially. We followed the “no touch principle” strictly in produce of operation, avoiding extrusion to the tumor and blocking the vessels to supply tumor firstly. But it was still impossible completely to prevent some cancer cells dropping from the tumor mass and entering into the vascular or lymphatic vessels. So, we presumed that those escaped cancer cells could implant in suit or in migration to metastatic sites to survive and to proliferate. These might be reasons for tumor recurrence and metastasis after operation, about which the furthermore researches should be taken on.

The ROC curve has been extensively used to evaluate the diagnostic accuracy and can also be used to judge the predictive value of the scoring system [24, 30]. By using the ROC curve in the current study to determine the cutoff for the BSV of CD133 mRNA was proved to be an effective prognostic factor in GC. Postsurgery survival time was shorter in patients with higher preoperative CD133 or postoperative mRNA expression in PBMCs in comparison with that in lower preoperative CD133 or postoperative CD133 mRNA expression.

It is well known that GC is a highly malignant tumor with a poor prognosis, especially when LNM exists. Early diagnosis for GC or for metastatic status of GC is the most important way for the early treatment and the suitable choose of therapy in order to improve the prognosis. Hence, the simple and less traumatic methodology means to monitor the metastasis and the recurrence is worth of the attempts to the early diagnosis to LNM and recurrence of GC. So CD133, a new diagnostic and therapeutic target, seems to be a potential prosperity for the clinical evaluation and the treatment of GC in future.

## 5. Conclusion

The level of CD133 mRNA was significantly elevated in PBMCs of GC in comparison with that in GU or in healthy volunteers, which was also associated with LNM, deeper invasion, later stages of TNM, and higher MLR. The higher level of CD133 mRNA in PBMCs, especially detected postoperatively, might predict the worse prognosis of GC. Patients who developed a higher level of preoperative or postoperative CD133 mRNA expression suffered a poorer survival.

## Figures and Tables

**Table 1:** The average BSV of preoperative CD133 mRNA in PBMCs correlated on the clinicopathological features.

Parameters	*n* (%)	Mean ± SD	Statistic	*P* value
Gender				
Male	45 (64.29%)	0.273 ± 0.160	*Z* = −0.441	0.659
Female	25 (35.71%)	0.242 ± 0.126		
Size of tumor (cm)				
<5	31 (44.29%)	0.238 ± 0.144	*Z* = −1.023	0.306
≥5	39 (55.71%)	0.281 ± 0.152		
Lauren type				
Intestinal type	54 (77.14%)	0.258 ± 0.155	*Z* = −0.700	0.484
Diffuse type	16 (22.86%)	0.276 ± 0.129		
Histological type				
1	23 (32.86%)	0.191 ± 0.102	*χ* ^2^ = 9.903	0.007
2	15 (21.43%)	0.274 ± 0.176		
3	32 (45.71%)	0.308 ± 0.148		
Vascular invasion				
Negative	49 (70.00%)	0.245 ± 0.145	*Z* = −1.244	0.214
Positive	21 (30.00%)	0.302 ± 0.154		
Lymphatic vessel invasion				
Negative	33 (47.14%)	0.210 ± 0.133	*Z* = −2.737	0.006
Positive	37 (52.86%)	0.308 ± 0.149		
Invasive depth				
*T*_1-2_	30 (42.86%)	0.200 ± 0.141	*Z* = −2.939	0.003
*T*_3-4_	40 (57.14%)	0.309 ± 0.139		
Lymph node metastasis				
Negative	28 (40.00%)	0.190 ± 0.135	*Z* = −3.748	<0.001
Positive	42 (60.00%)	0.310 ± 0.136		
TNM stage				
I-II	30 (42.86%)	0.191 ± 0.139	*Z* = −3.657	<0.001
III-IV	40 (57.14%)	0.315 ± 0.135		

**Table 2:** Multivariate risk analysis on the correlation of the BSV of preoperative CD133 mRNA in PBMCs with clinicopathological features.

Parameters	*B*	S.E.	Wald	df	Sig.	Exp(*B*)	95.0% of CI	
							Lower	Upper
Gender	−0.979	0.929	1.109	1	0.292	0.376	0.061	2.323
Size of tumor	−0.550	0.840	0.429	1	0.513	0.577	0.111	2.995
Lauren type	−2.268	1.393	2.651	1	0.103	0.104	0.007	1.587
Histological type	3.653	1.439	8.532	2	0.074	0.120	0.012	0.877
Vascular invasion	−1.206	1.124	1.150	1	0.284	0.299	0.033	2.713
Lymphatic vessel invasion	0.789	1.291	0.373	1	0.541	0.454	0.036	5.707
Invasive depth	3.531	1.561	0.116	1	0.034	1.700	0.080	36.268
Lymph node metastasis	0.785	1.560	0.254	1	0.615	2.193	0.013	1.293
TNM stage	0.915	2.266	0.163	1	0.686	2.497	0.029	211.924
CD133 mRNA in primary tissue	5.846	3.292	3.154	1	0.076	0.003	0.000	1.833
CD133 protein expressed in primary tissue	0.943	1.049	0.809	1	0.369	2.568	0.329	20.058
MLR	0.101	0.058	3.033	1	0.082	0.904	0.807	1.013
Metastatic lymph node number	0.647	0.290	4.968	1	0.026	1.190	1.081	3.375
Dissected lymph node number	−0.055	0.082	0.449	1	0.503	0.947	0.806	1.111

**Table 3:** The average BSV of postoperative CD133 mRNA in PBMCs correlated on the clinicopathological features.

Parameters	*n* (%)	Mean ± SD	Statistic	*P* value
Gender				
Male	45 (64.29%)	0.325 ± 0.170	*Z* = −0.545	0.585
Female	25 (35.71%)	0.293 ± 0.157		
Size of tumor (cm)				
<5	31 (44.29%)	0.282 ± 0.173	*Z* = −1.413	0.158
≥5	39 (55.71%)	0.338 ± 0.157		
Lauren type				
Intestinal type	54 (77.14%)	0.311 ± 0.172	*Z* = −0.657	0.511
Diffuse type	16 (22.86%)	0.321 ± 0.145		
Histological type				
1	23 (32.86%)	0.255 ± 0.149	*χ* ^2^ = 4.625	0.099
2	15 (21.43%)	0.315 ± 0.198		
3	32 (45.71%)	0.354 ± 0.152		
Vascular invasion				
Negative	49 (70.00%)	0.293 ± 0.157	*Z* = −1.570	0.116
Positive	21 (30.00%)	0.360 ± 0.178		
Lymphatic vessel invasion				
Negative	33 (47.14%)	0.252 ± 0.145	*Z* = −2.812	0.005
Positive	37 (52.86%)	0.367 ± 0.165		
Invasive depth				
*T*_1-2_	30 (42.86%)	0.215 ± 0.125	*Z* = −4.237	<0.001
*T*_3-4_	40 (57.14%)	0.387 ± 0.154		
Lymph node metastasis				
Negative	28 (40.00%)	0.228 ± 0.134	*Z* = −3.471	0.001
Positive	42 (60.00%)	0.370 ± 0.161		
TNM stage				
I-II	30 (42.86%)	0.211 ± 0.129	*Z* = −4.439	<0.001
III-IV	40 (57.14%)	0.389 ± 0.148		

**Table 4:** Multivariate risk analysis on the correlation of the BSV of postoperative CD133 mRNA in PBMCs with clinicopathological features.

Parameters	*B*	S.E.	Wald	df	Sig.	Exp(B)	95.0% of CI	
							Lower	Upper
Gender	0.056	0.870	0.004	1	0.948	1.058	0.192	5.817
Size of tumor	−1.309	0.955	1.877	1	0.171	0.270	0.042	1.757
Lauren type	0.794	1.368	0.338	1	0.561	2.212	0.152	32.196
Histological type	1.780	1.230	3.812	2	0.149	0.332	0.010	2.322
Vascular invasion	−0.525	1.104	0.226	1	0.634	0.592	0.068	5.151
Lymphatic vessel invasion	3.487	1.133	3.899	1	0.033	0.096	0.011	0.831
Invasive depth	5.149	1.227	3.069	1	0.018	8.580	0.775	95.016
Lymph node metastasis	0.917	1.605	0.327	1	0.568	2.502	0.108	58.140
TNM stage	2.680	2.035	1.734	1	0.188	14.587	0.270	787.336
CD133 mRNA in primary tissue	4.842	2.885	2.818	1	0.093	0.008	0.000	2.251
CD133 protein expressed in primary tissue	0.951	1.021	0.867	1	0.352	2.588	0.350	19.159
MLR	−0.084	0.054	2.411	1	0.121	0.919	0.826	1.022
Metastatic lymph node number	0.445	0.264	2.847	1	0.092	1.562	0.931	2.619
Dissected lymph node number	−0.134	0.078	2.987	1	0.084	0.875	0.751	1.018
